# ‘Veni, Vidi, Vaccini’: consensus-based vaccination pathway implementation in a paediatric tertiary hospital in Tuscany, Italy

**DOI:** 10.1093/eurpub/ckag109

**Published:** 2026-06-24

**Authors:** Walter Maria Sarli, Matilde Peri, Clementina Canessa, Francesca Lippi, Lorenzo Lodi, Francesca Menegazzo, Francesco Puggelli, Chiara Azzari, Silvia Ricci, FRAGI-le (Frontline Response Against Gaps in Immunisation) Group, Federica Barbati, Elisa Buti, Chiara Caparrelli, Paolo Del Greco, Donata Dini, Silvia Favilli, Grazia Fenu, Giacomo Folini, Valentina Guarnieri, Andrea La Tessa, Emanuela Laudani, Ilaria Maccora, Maria Vincenza Mastrolia, Federico Melani, Sonia Muricci, Marco Moroni, Silvia Passantino, Elena Procopio, Sara Renzo, Maria Chiara Sanvito, Luca Scarallo, Lisa Serafini, Gaia Spaziani, Annalisa Tondo, Sonia Toni, Irene Trambusti, Chiara Trapani, Francesca Trevisan, Gaia Varriale

**Affiliations:** Immunology Unit, Meyer Children’s Hospital IRCCS, Florence, Italy; Department of Health Sciences, University of Florence, Florence, Italy; Department of Health Sciences, University of Florence, Florence, Italy; Immunology Unit, Meyer Children’s Hospital IRCCS, Florence, Italy; Immunology Unit, Meyer Children’s Hospital IRCCS, Florence, Italy; Immunology Unit, Meyer Children’s Hospital IRCCS, Florence, Italy; Department of NEUROFARBA, University of Florence, Florence, Italy; Health Management Unit, Meyer Children’s Hospital IRCCS, Florence, Italy; Health Management Unit, Meyer Children’s Hospital IRCCS, Florence, Italy; Immunology Unit, Meyer Children’s Hospital IRCCS, Florence, Italy; Department of Health Sciences, University of Florence, Florence, Italy; Immunology Unit, Meyer Children’s Hospital IRCCS, Florence, Italy; Department of Health Sciences, University of Florence, Florence, Italy

## Abstract

Fragile paediatric patients are at higher risk of vaccine-preventable diseases, yet coverage remains suboptimal. Hospitalisation offers an opportunity to review and update immunisation, but fragmented practices create inequities, with access depending more on hospital context than clinical need. This study aimed to design, implement, and evaluate an in-hospital vaccination pathway for these patients. A mixed-methods approach was undertaken at Meyer Children’s Hospital IRCCS, Florence, Italy. Baseline assessment included nine focus groups and an online questionnaire with healthcare professionals from units managing fragile patients, exploring practices, barriers, and needs. Findings informed the co-design of a pathway integrating documentation review, catch-up planning, and in-hospital delivery coordinated by the Immunology Unit. Administrative data on non-SARS-CoV-2 vaccinations in fragile patients (2022–2025) were analysed. Most respondents supported hospital-based vaccination (94.2%) but reported gaps in registry access, spaces, and training. Only 67.6% collected vaccination history systematically, 14.4% always mentioned vaccination in discharge letters, and 15.9% always recommended family immunisation. Despite acknowledging low influenza coverage (60.9% declared coverage <50%), 15.9% expressed hesitancy in administering non-live vaccines, mainly due to efficacy concerns (55.9%). After implementation, vaccine administrations increased from 361 in the 2022–2023 season to 752 in the 2024–2025 season (+108.3%; *P* < .0001). Increases were observed after staff sensitisation, with further growth following the creation of the hospital vaccination centre. A structured hospital-based vaccination pathway for fragile paediatric patients is feasible, well accepted, and associated with increased vaccine administrations within the hospital care pathway. Broader adoption could reduce inequities and strengthen vaccine access for high-risk children.

## Introduction

Fragile paediatric patients represent a heterogeneous group that includes children with chronic conditions (e.g. cardio-respiratory, neurological, metabolic, neoplastic, and immunological diseases), severe disabilities (e.g. physical, sensory, intellectual, or mental), and those at greater risk of severe illness due to age (e.g. prematurity) [1]. These patients are not only more vulnerable to complications from vaccine-preventable diseases (VPDs), but also more exposed to infections because of their frequent contact with healthcare services, both in outpatient and inpatient settings [2]. While these patients represent a priority target for national and international public health [3], their clinical complexity often results in a fragmented preventive approach.

Immunisation coverage in fragile paediatric populations remains suboptimal. Uptake of recommended booster vaccines—including pneumococcal or meningococcal vaccines—may fall to around 40% in high-risk categories [4], while incomplete vaccination status affects up to half of adolescents with chronic conditions [5]. Coverage is even more critical for seasonal immunisation: European studies show that influenza vaccination reaches only 34% of children with chronic diseases [6], with Italian estimates ranging between 17.5% and 25%, well below recommended thresholds [7, 8].

This ‘protection gap’ is partly rooted in the organisational structure of the Italian healthcare system and the fragmentation of professional competences. Vaccination governance is divided across multiple institutional levels that often lack formal integration. Primary Care Paediatricians act as the first coordinators of child health playing a key role in counselling and promoting vaccination uptake, although vaccine administration is often delivered through dedicated vaccination services. These services are typically organised within the Departments of Prevention of Local Health Authorities (ASL), which hold primary responsibility for implementing immunisation programmes and maintaining vaccination registries. Hospitals may also contribute to vaccination delivery, particularly for patients with complex medical conditions requiring specialist follow-up. However, current hospital-based vaccination models, where they exist, are often disconnected from the institutional vaccination registry and the territorial prevention network [9]. Moreover, the regionalised nature of the Italian healthcare system further contributes to highly variable implementation, resulting in substantial variability in vaccine provision.

This separation leads to missed opportunities for immunisation. Community-based services may lack the clinical capacity to safely manage immunisations in children with severe comorbidities, while primary care providers may hesitate to authorise procedures without a formal specialist authorisation. Consequently, fragile children often fall through the cracks of the system. As a result, children who require the highest level of protection due to clinical vulnerability may remain among the least protected.

The latest Italian National Immunisation Plan (PNPV 2023–2025), aligned with the World Health Organization (WHO) Immunisation Agenda 2030, mandates a paradigm shift, identifying hospitals as strategic hubs for identifying and immunising high-risk groups [3, 10]. The plan explicitly urges the integration of hospital specialists and public health units to ensure that clinical complexity is no longer a barrier to prevention. International and national evidence, indeed, suggests that hospitalisation represents a strategic opportunity to assess and improve immunisation status, especially for children with chronic conditions [11]. It has been demonstrated that children with a catch-up vaccination plan show higher vaccination rates within 30–90 days post-admission, whereas nearly half without a plan remain overdue after 90 days [12].

However, hospital potential is frequently undermined by multiple barriers, including complex clinical pathways, competing health priorities, and gaps in communication between families and healthcare professionals [13]. Hospital workflows often prioritise acute management over preventive care, with immunisation perceived as secondary. Many hospitals also lack the infrastructure to support in-hospital catch-up vaccination, including trained staff, vaccine storage facilities, and dedicated organisational pathways. Furthermore, the absence of systematic staff training in vaccine counselling and administration limits the ability of healthcare professionals to address parental hesitancy or proactively recommend vaccination.

A recent survey conducted by the Italian Association of Paediatric Hospitals (AOPI), which involved 14 hospitals, revealed that although most institutions report offering vaccines to inpatients through various means, no guidelines on vaccination in hospitals are yet available, leaving healthcare professionals with little direction on how best to incorporate vaccination into the clinical care of fragile children [14]. The need to develop structured and standardised approaches to hospital-based vaccination has therefore been widely acknowledged. What remains unexplored is how such models can be effectively designed and implemented within existing healthcare structures.

To bridge this gap, Meyer Children’s Hospital IRCCS, a tertiary paediatric centre, developed and implemented a structured, consensus-based vaccination pathway specifically targeting fragile paediatric patients and the specialists responsible for their care. This model was co-designed through a multidisciplinary process involving clinical departments, immunologists, and public health experts. The present study describes the design, implementation, and impact evaluation of a multidisciplinary quality improvement framework. By integrating qualitative barrier analysis with a structured service transformation, we aimed to provide a scalable and reproducible model for hospital-based immunisation, ensuring that clinical complexity is no longer a barrier to vaccine equity for high-risk paediatric populations.

## Methods

### Study setting and target population

This study was conducted at Meyer Children’s Hospital IRCCS, Florence, Italy, a tertiary paediatric centre with 226 beds across multiple specialties. The vaccination pathway specifically targeted paediatric patients with chronic or complex medical conditions (e.g. cardio-respiratory, neurological, metabolic) who were already under regular follow-up in the hospital’s specialised clinics. Eligibility was based on the patient’s ongoing care within these services; therefore, only patients referred through these specialised pathways were included in the project.

### Study design

This study was designed as a multidisciplinary Health Service Evaluation and Quality Improvement initiative. The primary objective was to transition from fragmented vaccination practices to a structured, institutionalised model for fragile paediatric populations. The project followed a mixed-methods implementation framework, structured in four sequential phases to ensure that the intervention was both evidence-based and tailored to the specific clinical needs of a tertiary care setting.

#### Phase 1—Barrier identification

A qualitative exploratory phase was carried out between January 2024 and July 2024 through focus groups [15] organised with selected clinical departments involved in the care of fragile children. Ten departments were invited based on their patient population and involvement in chronic disease management: Neonatal Intensive Care Unit, Neurology and Metabolic/Neuromuscular Units, Complex Care Units, Nephrology, Oncology/haematology, Gastroenterology, Diabetology, Rheumatology, Broncho-pneumology, and Cardiology. Nine focus group sessions were held with 4–8 participants, typically including the unit head, consultants, senior residents, and, when applicable, dedicated nurses. Each session lasted approximately 60 min and was conducted in person, guided by a semi-structured discussion guide. The discussions focused on current vaccination practices, perceived barriers, attitudes towards vaccination in fragile children, and suggestions for potential improvements. Sessions were moderated by trained facilitators, including immunologists. An inductive manual content analysis was performed on the transcripts to identify recurring themes, which were then categorised and synthesised in a report to inform the subsequent intervention design (see Supplementary Tables S1 and S2 in Appendix S1 for details).

#### Phase 2—Baseline validation

Following the focus group discussions, participants and other members of the same units were invited to complete a voluntary, anonymous, self-administered online questionnaire to complement and validate the insights that emerged during the meetings. The questionnaire included 19 items: 18 multiple-choice and one open-ended question. It was structured in two sections: the first collected anonymised demographic and professional information, while the second assessed perceptions of vaccine safety, perceived barriers, current practices, and unmet training needs related to immunisation in fragile children. The questionnaire is available in Supplementary Appendix S1. Insights gained from the questionnaire were used to inform and structure the discussions in subsequent focus group sessions.

#### Phase 3—Service implementation

Insights from the questionnaire and focus group discussions guided the development of enhanced intra-hospital communication strategies on vaccines. Moreover, these contributed to the design of a vaccination centre tailored to healthcare professionals’ needs. This centre was established in July 2024 to provide a structured pathway for fragile paediatric patients and ensure systematic immunisation. The vaccination centre remains active.

#### Phase 4—Impact evaluation

Vaccination activity data were collected from hospital administrative records covering July 2022 to June 2025, using the procedural code ‘injection of other prophylactic substances’ (International Classification of Diseases, 9th Revision, 99.29 code) which includes all non-SARS-CoV-2 paediatric vaccinations. Data were stratified by months and years. To ensure cohort specificity, the automated extraction was limited to codes linked to departments involved in the care of fragile patients, thereby excluding routine vaccinations administered to non-fragile populations. The analysis was therefore restricted to patients in regular chronic follow-up within these specialised units. Three seasons were analysed: July 2022–June 2023, July 2023–June 2024, July 2024–June 2025. GraphPad Prism 10 was used for statistical analysis. Data were first assessed for normality. For non-normally distributed data, the nonparametric Friedman test for paired data was applied, followed by Dunn’s multiple comparisons test to evaluate differences between individual years. Results were considered statistically significant at *P* < .05.

### Ethics

In accordance with national regulations and International Good Clinical Practice standards, formal approval from an Ethics Committee was not required. The quantitative analysis relied on de-identified, anonymised, and aggregated administrative data collected for institutional monitoring purposes. Focus groups were conducted with healthcare professionals as part of routine service mapping and pathway co-design activities. The online questionnaire was voluntary and anonymous, and no identifiable personal data were collected. All procedures followed the standard-of-care protocols of the Italian National Immunisation Plan (PNPV 2023–2025).

## Results

Between January 2024 and June 2024, a total of nine focus group meetings were conducted at Meyer Children’s Hospital IRCCS involving professionals from paediatric clinical units dealing with complex and medically fragile patients. The sessions engaged 47 participants, including 57.4% consultants, 38.3% residents, and 4.3% nurses.

The discussions revealed widespread awareness of the increased risk posed by vaccine-preventable diseases in medically fragile populations, and strong interest in improving vaccination strategies within the hospital setting. All units expressed the need for systematic approaches to identify under-vaccinated patients, integrate immunisation into routine clinical care, and improve documentation and registry reporting. All units requested a standard discharge statement prompting vaccination review in electronic clinical reports, as well as posters and leaflets for staff, whereas almost all units (8/9 focus groups) agreed with dedicated vaccination slots, a vaccine section in clinical records, a focus on recommended vaccines, influenza vaccination in the ward, the request for vaccination documents at hospitalisation, extended screening serologies before immunosuppressive treatments, and standardised protocols (see Supplementary Appendix S1 for detailed focus group discussion summary).

At the conclusion of the focus groups, an online questionnaire was distributed and completed not only by participants but also by additional medical and nursing staff from the involved units, yielding a total of 69 responses. Nearly half of the respondents (43.5%) had fewer than 5 years of professional experience, while 17.4% reported 5–10 years, and 39.1% had more than a decade in practice. In terms of professional roles, medical residents accounted for 46.4% of the sample, medical consultants for 39.1%, and nurses for 14.5%.

Only 23.2% reported regularly administering vaccinations in hospital, while 71% considered hospital vaccinations appropriate only in selected cases. Vaccinations in hospital were regularly performed in consultation with Immunology services in 59.7% of cases, whereas only 32.8% administered vaccinations within their own hospital unit.

**Figure 1.**
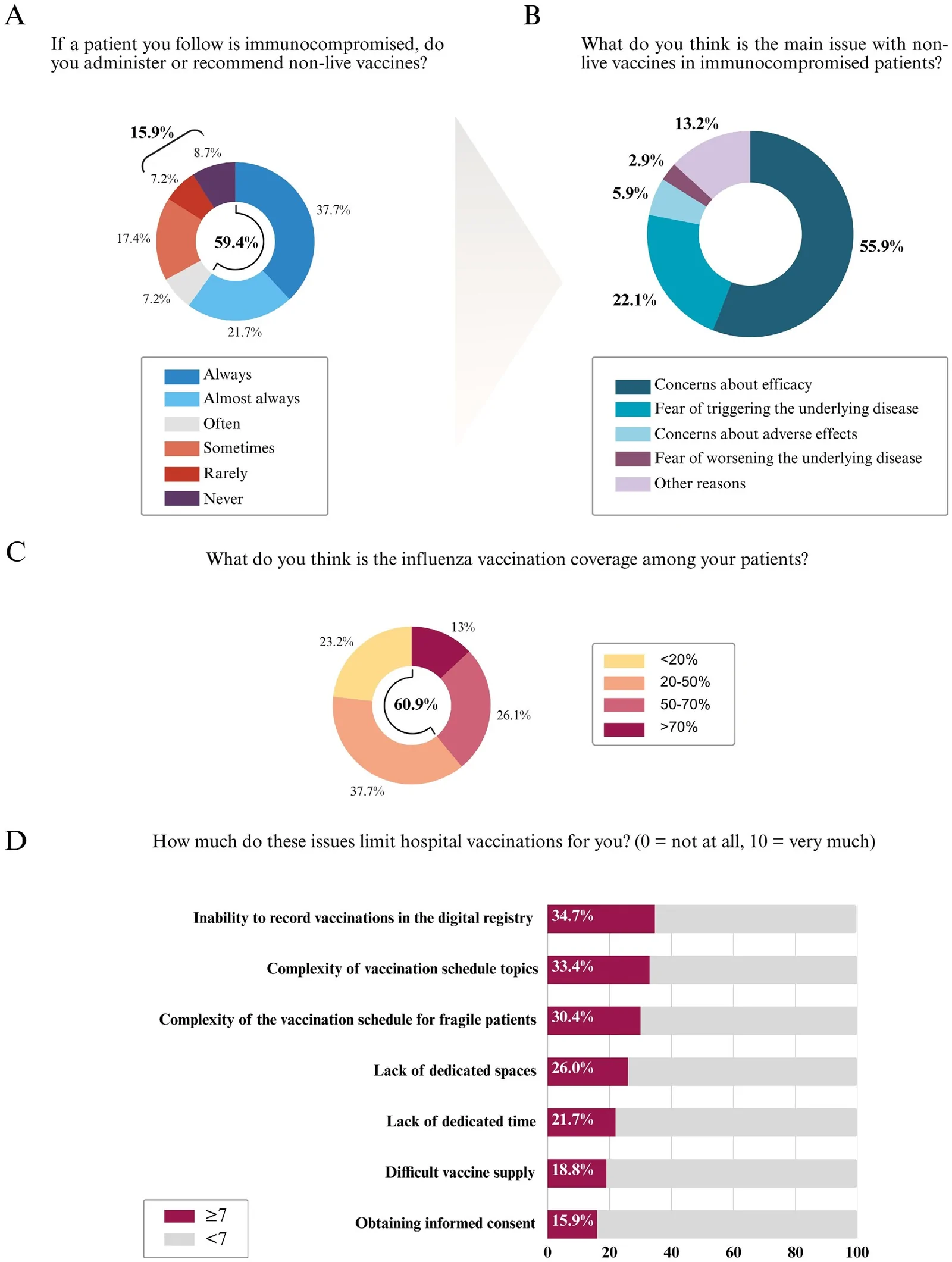
Healthcare professionals’ practices and perceptions regarding vaccinations in immunocompromised patients and influenza coverage. Panel A shows the frequency of administering or recommending non-live vaccines in immunocompromised patients. Panel B illustrates the main perceived issues with non-live vaccines in these patients. Panel C reports estimated influenza vaccination coverage among patients. Panel D shows the main perceived challenges related to administering vaccinations to fragile patients in hospital settings. Percentages represent the distribution of responses; in Panel D, they indicate the proportion of responses scoring ≥7, corresponding to high perceived relevance.

The responses to the questions are presented in detail in Table 1 and Fig. 1.

**Table 1.** Selected questions on hospital-based vaccination practices and attitudes of healthcare professionals.^a^

Do you think that vaccine-preventable diseases may play a role in influencing the course of the conditions you manage?	
≥7	77.8%
<7	22.2%
Do you think that vaccine-preventable diseases have a more severe course in fragile patients?	
≥7	92.4%
<7	7.6%
Do you collect information on previous vaccinations in the medical history?	
Always	67.6%
Only in selected cases	17.6%
Never	11.8%
Do not know	3.0%
In discharge letters, do you indicate missing vaccinations and how to catch up on them?	
Always or almost always	14.4%
Often	18.8%
Sometimes	14.5%
Rarely or never	52.3%
Do you discuss missing vaccinations for family members?	
Always or almost always	15.9%
Often	18.8%
Sometimes	27.6%
Rarely or never	37.7%
Do you think it would be useful to have a dedicated vaccination section in clinical records?	
≥7	95.7%
<7	4.3%
Do you think it is important to check whether a fragile patient has received all recommended vaccinations?	
≥7	95.5%
<7	4.5%
Do you think a fragile patient would benefit from receiving vaccination in hospital during a specialist follow-up visit?	
≥7	92.7%
<7	7.3%

a Percentages indicate the distribution of responses. For questions scored on a 0–10 scale, values ≥7 indicate agreement, with 0 representing complete disagreement and 10 complete agreement. The table highlights practices related to fragile patients, collection of vaccination history, communication in discharge letters, and perceived benefits of in-hospital vaccination.

Based on the barriers shown in Fig. 1, Panel D, 94.2% of respondents considered a hospital vaccination centre, responsible for all vaccinations for fragile patients, to be very useful (≥7).

In response to these findings, a hospital-wide vaccination pathway was designed and implemented in early 2024. The pathway was based on the systematic integration of vaccination assessment into the care of fragile patients, supported by centralised immunological oversight.

Starting in July 2024, a dedicated vaccination centre was thus created, staffed by seven physicians from the Immunology Unit, with the support of a professional nurse who administers the vaccines. The service offers scheduled vaccine appointments within the hospital’s day-care system, for two afternoon shifts per week, while on the remaining days vaccinations are provided directly by the Immunology Service. The centre is also structured to accommodate patients referred from other departments on a walk-in basis in case of urgent vaccination needs, such as during influenza outbreaks or when vaccination windows are limited.

**Figure 2.**
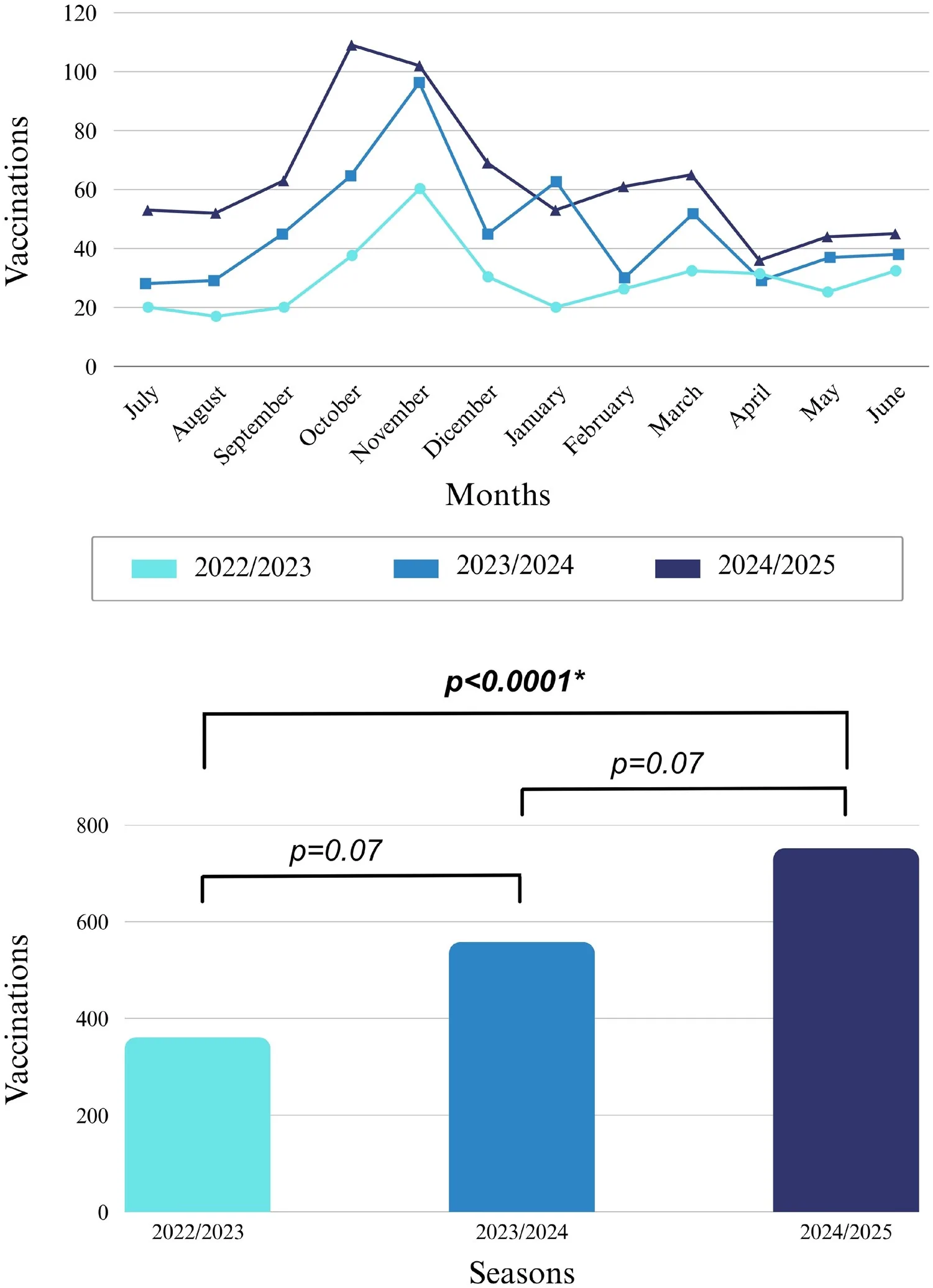
Monthly vaccination trends across three consecutive seasons: 2022–2023, 2023–2024, and 2024–2025. The line graph shows monthly vaccine administrations across the three seasons. The bar graph displays the total number of vaccinations per season, showing a statistically significant increase in 2024–2025 compared with 2022–2023 (*P* < .0001). The observed increase occurred during staff sensitisation activities and following the establishment of a dedicated hospital vaccination centre.

To evaluate the potential impact of the intervention, administrative data on vaccine administration were extracted from the hospital records using the procedure code ‘injection of other prophylactic substances’. Vaccination numbers for the 2024–2025 season were compared with previous years. A total of 361 vaccines were administered in 2022–2023, 558 in 2023–2024, and 752 in 2024–2025. This corresponds to a 54.6% increase in total vaccinations in 2023–2024 compared to 2022–2023, and a further 34.8% increase in 2024–2025 compared to 2023–2024. Overall, the 2024–2025 season saw a 108.3% increase in total vaccinations relative to 2022–2023. Data are illustrated in Fig. 2.

## Discussion

This study describes the design, implementation, and preliminary outcomes of a hospital-based vaccination pathway targeting fragile paediatric patients at Meyer Children’s Hospital IRCCS. Our findings support the role of hospital encounters as an opportunity to assess immunisation status and facilitate access to recommended vaccines among fragile paediatric patients, consistent with previous evidence highlighting inpatient settings as strategic points for preventive care [11, 12].

### Hospital vaccination as a strategic opportunity

Baseline data from the focus groups and online questionnaire revealed substantial heterogeneity in vaccination practices, both in vaccine offer and integration into clinical workflows. Similar to prior studies [9, 14, 16], barriers were multifactorial, including organisational issues (e.g. absence of standardised protocols, fragmented documentation systems), resource limitations (e.g. dedicated spaces and time), and misconceptions about vaccine safety and efficacy in medically complex children. It has been observed that up to one in five inpatients are incompletely immunised [17], a figure likely underestimated, particularly among fragile patients who often have incomplete or insufficient vaccination records. While most hospital records, indeed, may contain some immunisation data, a significant proportion (up to 69%) remains generic or incomplete. Many children are mislabelled as ‘up-to-date with vaccination’ without proper verification or when only mandatory vaccines have been documented [17]. Such inertia is further confirmed in vulnerable adult populations, where studies indicate that over 80% of patients with a clear clinical indication for vaccination are not correctly protected at the time of admission [18].

At our centre, in 2022, a survey of 178 families focusing on influenza vaccination in fragile children confirmed critical gaps, with nearly half reporting that their child had not received seasonal influenza vaccination in previous years, often due to the absence of a direct recommendation from healthcare providers (43.2%). Furthermore, parental perception of influenza as a serious health risk was low, despite the presence of chronic conditions [19]. Consistent with previous literature, parents reported higher rates of missed vaccinations due to frequent acute illnesses and hospital admissions [16]. Our findings aligned with previous experiences where targeted provider education and awareness in specialty clinics were shown to be essential to overcome missed opportunities and significantly improve vaccination rates for specific pathogens, such as pneumococcus [20].

The implementation of the ‘Veni, Vidi, Vaccini’ pathway was associated with a 108.3% increase in vaccine administrations, indicating that hospital-based strategies may help reduce missed opportunities for vaccination in medically fragile children. Current literature supports this approach and shows that opportunistic hospital-based vaccination can reach significantly higher coverage (up to 71% for routine vaccines) compared to community referral alone [11]. However, as noted in recent scoping reviews, the primary challenge remains the rapid verification of immune status, a barrier that can be overcome through centralised coordination and the use of interconnected electronic registries [21].

### Healthcare professional perceptions

Our 2022 survey showed that over 80% of parents expressed confidence in vaccine safety, and many would reconsider their decision if advised by a trusted specialist, suggesting that families are open to vaccination when supported by clear and trusted guidance [19]. Other studies report that both parents (89%) and healthcare professionals (87%) consider opportunistic inpatient vaccination acceptable [16].

This is consistent with international observations where parents of hospitalised children generally hold favourable attitudes towards inpatient vaccination and often disagree with the provider’s perception that the child is ‘too sick’ to be vaccinated during the stay [16, 22]. Specifically, while clinical success is strongly linked to parental trust in vaccine safety and the recognition of the need for annual dosing, a significant misalignment often exists between families and providers [22]. Focus groups and the follow-up questionnaire highlighted that healthcare professionals recognise the importance of vaccination but face ‘competing priorities’. Interestingly, while the vast majority of providers acknowledge that inpatient vaccination is a priority, they frequently misidentify parental reluctance as the main barrier [22]. In reality, our data and the literature suggest that organisational failures—such as staff simply forgetting to screen the vaccination status or order the dose—are more prevalent than actual parental refusal [22]. Although 60.9% recognised that influenza coverage was below 50%, staff still reported hesitancy in administering non-live vaccines to fragile patients (15.9%), mainly due to concerns about efficacy (55.9%). This ‘clinical inertia’ in specialty units, particularly in oncology/haematology, can be addressed by providing the specialised immunological clearance and multidisciplinary support identified as key facilitators in complex clinical settings [23]. As demonstrated in similar paediatric hospital interventions, the direct involvement and sensitisation of the clinical staff are the primary drivers for the success of such pathways [20]. Furthermore, theoretical models such as ‘Capability, Opportunity, Motivation, and Behaviour’ (COM-B) highlight that healthcare professionals’ capability and motivation are the true drivers of organisational change [24]. By providing formal specialist endorsement, the ‘trust and knowledge gap’ often identified in the literature can be effectively bridged, transitioning the specialist from a passive observer to an active advocate [13].

### Impact of the intervention

The implemented vaccination pathway addressed these gaps by combining staff sensitisation, enhanced communication, and the creation of a dedicated vaccination centre, coordinated by the Immunology Unit. The establishment of the hospital’s new vaccination centre in July 2024 was one of the key outcomes of the initiative and represents an institutional step towards the integration of vaccine services into routine hospital care. A striking result of this intervention is the 54.6% increase in vaccinations recorded during the initial sensitisation and focus group phase, prior to the physical opening of the vaccination centre. This suggests that sensitisation and focus group engagement alone can positively influence practice. By involving multiple clinical departments in a co-design process to address logistical needs (e.g. standardised discharge templates), the intervention aligns with the ‘Be inFLUential’ model, which suggests that hands-on, multi-step approaches are more effective than top-down orders [25]. By integrating vaccination into established clinical pathways, the efficiency of every specialist visit is maximised. This quality improvement process is proven to raise immunisation rates even in highly complex populations, as seen in models where rates increased from 20% to 60% through continuous monitoring and peer support [12]. Following the establishment of the vaccination centre, providing ready access, dedicated timeslots, and specialist supervision, vaccine administrations increased further. While causality cannot be definitively established in the absence of a control group, trends indicate that structural interventions can rapidly improve access to immunisation for high-risk populations. Part of this improvement may also reflect heightened awareness among healthcare staff generated during preparatory phases of the project, including focus group discussions and dissemination of preliminary findings. Over the 2-year period, total vaccine administrations among fragile patients increased by more than 100%, supporting the potential value of staff education and structural facilitation.

Nevertheless, this project has limitations. Only ten departments were initially involved in the co-design of the pathway, selected based on their management of large numbers of immunocompromised or medically complex children, potentially limiting generalisability across the entire hospital. The focus group methodology generated qualitative data only. In addition, the questionnaire provided qualitative and self-reported data, which precluded more extensive statistical analysis of perceptions. Nevertheless, the large magnitude of change and consistency with staff-reported barriers and facilitators support the plausibility and practical relevance of the intervention.

## Conclusion

This project demonstrated that, with limited resources, national and international recommendations on vaccination for medically fragile children can be translated into practice within a tertiary hospital setting. By involving key departments through focus groups and structured consultation, we identified barriers and co-designed practical solutions. Fragmented practices may contribute to inequities, whereby a child’s access to timely vaccination depends more on the hospital or region of residence than on clinical need. In this context, the lack of clear guidance and institutional models represents a barrier to both equity and efficiency in the delivery of preventive care. Our initiative increased healthcare professional awareness and was associated with a substantial increase in vaccine administrations within the hospital pathway. The creation of an in-hospital vaccination centre represents a concrete organisational step towards improving access to recommended immunisation for fragile children. While initially limited to selected services, the model now allows hospital-wide patient referral. Nonetheless, this experience may serve as a practical framework for other paediatric hospitals aiming to reduce missed opportunities and improve vaccine accessibility.

## Supplementary Material

ckag109_Supplementary_Data

## Data Availability

The original contributions presented in the study are included in the article. Further inquiries can be directed to the corresponding author. Key points Fragile paediatric patients remain insufficiently protected against vaccine-preventable diseases despite frequent healthcare contact. Hospitalisation represents an opportunity to assess immunisation status and facilitate vaccine delivery. A dedicated hospital vaccination centre may increase vaccine administrations and improve staff awareness. Institutional commitment and clear clinical protocols are essential to ensure equitable vaccination for fragile children.
