# Supplementary material for: ‘Veni, Vidi, Vaccini’: consensus-based vaccination pathway implementation in a paediatric tertiary hospital in Tuscany, Italy
Source: Eur J Public Health. 2026 Jun 24;36(4):ckag109. doi: 10.1093/eurpub/ckag109 (PMC13293060; doi:10.1093/eurpub/ckag109)
Supplement: ckag109_Supplementary_Data [file ckag109_supplementary_data.zip › ejph-2025-10-om-0891-File005.docx]

**FRAGI-le (Frontline Response Against Gaps in Immunisation) Group**

Federica Barbati^1^, MD, Elisa Buti^2^, MD, Chiara Caparrelli^3^, MD, Paolo Del Greco^3^, MD, Donata Dini^4^, RN, Silvia Favilli^5^, MD, Grazia Fenu^3^, MD, Giacomo Folini^6^, Valentina Guarnieri^1^, MD, Andrea La Tessa^2^, MD, Emanuela Laudani^7^, MD, Ilaria Maccora^8^, MD, Maria Vincenza Mastrolia^8^, MD, Federico Melani^9^, MD, Sonia Muricci^4^, RN, Marco Moroni^10^, MD, Silvia Passantino^5^, MD, Elena Procopio, MD^12^, Sara Renzo^11^, MD, Maria Chiara Sanvito^13^, MD, Luca Scarallo^11^, MD, Lisa Serafini^10^, MD, Gaia Spaziani^5^, MD, Annalisa Tondo^13^, MD, Sonia Toni^7^, MD, Irene Trambusti^13^, MD, Chiara Trapani^4^, MD, Francesca Trevisan^13^, MD, Gaia Varriale^7^, MD

^1^ Immunology Unit, Meyer Children’s Hospital IRCCS, Florence, Italy

^2^ Nephrology and Dialysis Unit, Meyer Children’s Hospital IRCCS, Florence, Italy

^3^ Paediatric Pulmonary Unit, Meyer Children’s Hospital IRCCS, Florence, Italy

^4^ Department of Paediatrics, Meyer Children's Hospital IRCCS, Florence, Italy

^5^ Department of Paediatric Cardiology, Meyer Children's Hospital IRCCS, Florence, Italy

^6^ Financial and Management Control, Meyer Children's Hospital IRCCS, Florence, Italy

^7^ Endocrinology and Diabetology Unit, Meyer Children's Hospital IRCCS, Florence, Italy

^8^ Rheumatology Unit, ERN ReCONNET Center, Meyer Children's Hospital IRCCS, Florence, Italy

^9^ Neuroscience Department, Meyer Children's Hospital IRCCS, Florence, Italy

^10^ Neonatal Intensive Care Unit, Meyer Children's Hospital IRCCS, Florence, Italy

^11^ Gastroenterology and Nutrition Unit, Meyer Children's Hospital IRCCS, Florence, Italy

^12^ Metabolic and Neuromuscular Unit, Meyer Children's Hospital IRCCS, Florence, Italy

^13^ Division of Paediatric Oncology/Haematology, Meyer Children's Hospital IRCCS, Florence, Italy

**Supplementary Box S1. Detailed online questionnaire**

*The questionnaire included 19 items: 18 multiple-choice and one open-ended question. It was structured in two sections: the first collected anonymised demographic and professional information, while the second assessed perceptions of vaccine safety, perceived barriers, current practices, and unmet training needs related to immunisation in fragile children.*

| **QUESTION** | **RESPONSES** |
| --- | --- |
| **Years of professional experience** | <5, 5-10, >10 |
| **Affiliated Specialty Unit** | Neonatal Intensive Care Unit, Neurology and Metabolic Diseases, Complex Care Unit Nephrology, Oncohaematology, Gastroenterology, Diabetology, Rheumatology, Bronchopneumology, and Cardiology |
| **Role** | Consultant, Resident, Nurse |
| **Do you think that vaccine-preventable diseases may play a role in influencing the course of the conditions you manage?** | 0–10 scale (with 0 representing complete disagreement and 10 complete agreement) |
| **Do you think that vaccine-preventable diseases have a more severe course in fragile patients?** | 0–10 scale (with 0 representing complete disagreement and 10 complete agreement) |
| **If a patient you follow is immunocompromised, do you administer or recommend non-live vaccines?** | Always, Almost always, Often, Sometimes, Rarely, Never |
| **What do you think is the main issue with non-live vaccines in immunocompromised patients?** | Concerns about efficacy, Fear of triggering the underlying disease, Concerns about adverse effects, Fear of worsening the underlying disease, Other reasons |
| **What do you think is the influenza vaccination coverage among your patients?** | <20%, 20-50%, 50-70%, >70% |
| **Do you think it is important to check whether a fragile patient has received all recommended vaccinations?** | 0–10 scale (with 0 representing complete disagreement and 10 complete agreement) |
| **Do you think a fragile patient would benefit from receiving vaccination in hospital during a specialist follow-up visit?** | 0–10 scale (with 0 representing complete disagreement and 10 complete agreement) |
| **Do you collect information on previous vaccinations in the medical history?** | Always, Only in selected cases, Never, Don’t know |
| **In discharge letters, do you indicate missing vaccinations and how to catch up on them?** | Always, Almost always, Often, Sometimes, Rarely, Never |
| **Do you discuss missing vaccinations for family members?** | Always, Almost always, Often, Sometimes, Rarely, Never |
| **Do you think it would be useful to have a dedicated vaccination section in clinical records?** | Yes, No, Don’t know |
| **When planning the management pathway for an individual patient, do you consider administering vaccinations in the hospital?** | Always, Only in selected cases, Never |
| **If yes, where are vaccinations administered?** | In your own department, On advice from the Immunology Service, During hospital admission on advice, Both situations, depending on the case |
| **How much do these issues limit hospital vaccinations for you? (Inability to record vaccinations in the digital registry, Complexity of vaccination schedule topics, Complexity of the vaccination schedule for fragile patients, Lack of dedicated spaces, Lack of dedicated time, Difficult vaccine supply, Obtaining informed consent)** | 0–10 scale (with 0 representing complete disagreement and 10 complete agreement) |
| **Do you think a dedicated Vaccination Centre managing all vaccines for all fragile patients would be useful in the hospital?** | Yes, No, Don’t know |
| **Any suggestions?** | Open-ended answer |
