# Supplementary material for: ‘Veni, Vidi, Vaccini’: consensus-based vaccination pathway implementation in a paediatric tertiary hospital in Tuscany, Italy
Source: Eur J Public Health. 2026 Jun 24;36(4):ckag109. doi: 10.1093/eurpub/ckag109 (PMC13293060; doi:10.1093/eurpub/ckag109)
Supplement: ckag109_Supplementary_Data [file ckag109_supplementary_data.zip › ejph-2025-10-om-0891-File006.docx]

Table S1

| **Focus Group** | **Context (summary)** | **Proposals (summary)** |
| --- | --- | --- |
| **1st – Neonatal Intensive Care Unit** | Vaccinations during hospital stay; delays linked to clinical issues; no access to online registry; lack of vaccination indications at discharge; poor knowledge of complete schedule. | Continue inpatient vaccination; reduce delays; access to Regional Vaccination Registry; standard discharge sentence; posters/leaflets for personnel. |
| **2nd – Paediatric Neurology, Metabolic Diseases Unit, Clinically Complex Care Unit** | Vaccinations during hospital stay; no access to online registry; family mistrust; uncertainty on need of premedication/new vaccines; complex bookings; lack of vaccination indications at discharge; poor knowledge of complete schedule. | Request vaccination documents; focus on recommended vaccines; involve immunologist; standardised protocols; vaccination slots in day hospital; discharge sentence; vaccine section in clinical records; posters/leaflets for personnel. |
| **3rd – Paediatric Nephrology Day Hospital** | Vaccination history focused on mandatory; attention to transplant patients; problems with patients lacking documents; adolescents without caregiver in dialysis; absence of protocols for PCV15/20 and specific HBV; need for continuity and awareness. | Bring documentation; check recommended vaccines; plan vaccinations with guardian present; standardised protocols; vaccination slots in Day Hospital; discharge sentence; vaccine section in records; care pathway (DTCP); posters/leaflets for personnel. |
| **4th – Oncohaematology** | Pre-chemotherapy checklist limited to VZV/HBV; little control on recommended vaccines; difficulties with general practitioners for post-transplant schedule; poor influenza adherence; lack of discharge indications; need for awareness. | Extend serologies; request documentation; plan post-transplant vaccinations with immunologist; influenza vaccine in ward; vaccination slots in Day Hospital; discharge sentence; vaccine section in records; DTCP; posters/leaflets for personnel. |
| **5th – Paediatric Gastroenterology** | Limited vaccination evaluation; difficulties with timing for non-mandatory; low influenza adherence; limited appointment availability; lack of follow-up post-discharge; poor ward information; need for DTCP. | Extend serologies; request vaccination booklets; simplified schedules; direct vaccinations; vaccination slots in Day Hospital; discharge sentence; vaccine section in clinical records; posters/leaflets; internal survey for personnel. |
| **6th – Diabetology** | Evaluation limited to mandatory; timing difficulties; supply problems for influenza vaccine; few immunology slots; no vaccine section in records; need for dissemination; adult patients at risk of HZ; DTCP inclusion. | Check and recover vaccines; posters/comparison sheets; immediate slots; discharge sentence; temporary paper solution; posters/leaflets; VZV serology control and HZ vaccine; internal survey for personnel. |
| **7th – Paediatric Pneumology** | Vaccination checks not systematic; little attention to recommended vaccines; low influenza adherence; difficulty accessing hospital vaccinations; lack of discharge indications; poor ward information; DTCP inclusion. | Insert systematic vaccination screening; focus on recommended vaccines; influenza vaccinations in ward; vaccination slots in Day Hospital; discharge sentence; posters/leaflets for personnel; |
| **8th – Paediatric Rheumatology** | Vaccine checks only before biologics; little control on recommended vaccines; difficulty managing timing under immunosuppression; low influenza adherence; difficulty accessing slots; lack of discharge reminders; DTCP inclusion. | Extend screening to all vaccines; protocols for timing under immunosuppression; influenza vaccinations in ward; vaccination slots in Day Hospital; discharge sentence; posters/leaflets for personnel; |
| **9th – Paediatric Cardiology** | Poorly structured vaccination check; little attention to recommended vaccines; low influenza adherence; difficulty accessing vaccinations; lack of discharge reminders; need for DTCP. | Complete screening; focus on recommended vaccines; influenza vaccination in ward; vaccination slots in Day Hospital; discharge sentence; posters/leaflets for personnel. |

***Table S1****. Summary of findings from nine focus groups conducted across paediatric specialty units at Meyer Children’s Hospital. The left column reports the current context and main challenges in vaccination practices as described by participants; the right column outlines the proposals suggested by healthcare professionals to improve vaccination pathways for fragile patients. Legend: DTCP, Diagnostic, Therapeutic and Care Pathway; HBV, hepatitis B virus; HZ, herpes zoster; PCV, pneumococcal conjugate vaccine; VZV, varicella zoster virus.*

Table S2

| Number focus group /total focus group | Theme |
| --- | --- |
| 9/9 | Agreement on expanding the vaccination schedule for fragile patients. Need for improving integration of the digital health record.  Demand for more access to vaccination slots.  Request for enhanced training on vaccination protocols.  Importance of clear communication between vaccination centres and general paediatrician. Need for better data reporting tools. |
| 8/9 | Need for dedicated vaccination slots. Demand for vaccine section in clinical records. Request for focus on recommended vaccines. Agreement on influenza vaccination in the ward. Request for vaccination documents at hospitalisation Agreement on extended screening serologies. Need for standardised protocols |

***Table S2****. Shared themes emerging from the focus groups. Items reported as 9/9 indicate proposals unanimously supported across all groups, while items reported as 8/9 indicate proposals endorsed by the majority of groups.*
